# Discriminant analysis of principal components: a new method for the analysis of genetically structured populations

**DOI:** 10.1186/1471-2156-11-94

**Published:** 2010-10-15

**Authors:** Thibaut Jombart, Sébastien Devillard, François Balloux

**Affiliations:** 1 MRC Centre for Outbreak Analysis and Modelling, Department of Infectious Disease Epidemiology, Imperial College Faculty of Medicine, St Mary's Campus, Norfolk Place, London W2 1PG, UK; 2 Université de Lyon, Université Lyon1, UMR 5558 - LBBE "Biométrie et Biologie évolutive" Bât. Grégor Mendel, 43 bd du 11 novembre 1918, 69622 Villeurbanne cedex, France

## Abstract

**Background:**

The dramatic progress in sequencing technologies offers unprecedented prospects for deciphering the organization of natural populations in space and time. However, the size of the datasets generated also poses some daunting challenges. In particular, Bayesian clustering algorithms based on pre-defined population genetics models such as the STRUCTURE or BAPS software may not be able to cope with this unprecedented amount of data. Thus, there is a need for less computer-intensive approaches. Multivariate analyses seem particularly appealing as they are specifically devoted to extracting information from large datasets. Unfortunately, currently available multivariate methods still lack some essential features needed to study the genetic structure of natural populations.

**Results:**

We introduce the *Discriminant Analysis of Principal Components *(DAPC), a multivariate method designed to identify and describe clusters of genetically related individuals. When group priors are lacking, DAPC uses sequential K-means and model selection to infer genetic clusters. Our approach allows extracting rich information from genetic data, providing assignment of individuals to groups, a visual assessment of between-population differentiation, and contribution of individual alleles to population structuring. We evaluate the performance of our method using simulated data, which were also analyzed using STRUCTURE as a benchmark. Additionally, we illustrate the method by analyzing microsatellite polymorphism in worldwide human populations and hemagglutinin gene sequence variation in seasonal influenza.

**Conclusions:**

Analysis of simulated data revealed that our approach performs generally better than STRUCTURE at characterizing population subdivision. The tools implemented in DAPC for the identification of clusters and graphical representation of between-group structures allow to unravel complex population structures. Our approach is also faster than Bayesian clustering algorithms by several orders of magnitude, and may be applicable to a wider range of datasets.

## Background

The study of the genetic structure of biological populations has attracted a growing interest from a wide array of fields, such as population biology, molecular ecology, and medical genetics. One of the most widely applied approaches is the inference of population structuring with Bayesian clustering methods such as STRUCTURE [1,2] and BAPS [3,4]. These methods are particularly appealing as they allow for identifying genetic clusters under an explicit population genetics model. The popularity of these approaches leaves no doubt about their usefulness for extracting meaningful information from genetic data.

Unfortunately, the reliance of Bayesian clustering methods on explicit models also comes at a cost. Model-based approaches rely on assumptions such as the type of population subdivision, which are often difficult to verify and can restrict their applicability. Furthermore, estimation of a large number of parameters [5] can require considerable computational time when analyzing large datasets. To take full advantage of the increase in size and complexity of genetic datasets, fast and flexible exploratory tools are equally needed.

Multivariate analyses have been used for decades to extract various types of information from genetic data and have attracted renewed interest in the field [6-12]. In particular, principal component analysis (PCA) [13-15] has recently been suggested as an alternative to Bayesian clustering algorithms [5,11,12,16]. The main asset of PCA is its ability to identify genetic structures in very large datasets within negligible computational time, and the absence of any assumption about the underlying population genetic model.

**Figure 1:**
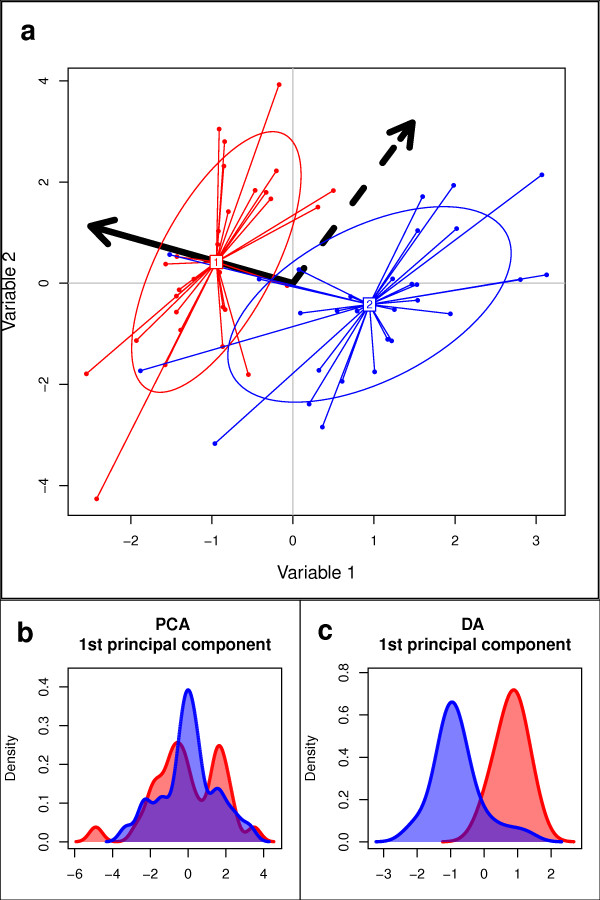
**Fundamental difference between PCA and DA**. (a) The diagram shows the essential difference between Principal Component Analysis (PCA) and Discriminant Analysis (DA). Individuals (dots) and groups (colours and ellipses) are positioned on the plane using their values for two variables. In this space, PCA searches for the direction showing the largest total variance (doted arrow), whereas DA maximizes the separation between groups (plain arrow) while minimizing variation within group. As a result, PCA fails to discriminate the groups (b), while DA adequately displays group differences.

However, PCA lacks some essential features for investigating the genetic structure of biological populations. First, it does not provide a group assessment, and would require *a priori *definition of clusters to study population structures. But even then, PCA would not be appropriate to obtain a clear picture of between-population variation (Figure 1). PCA aims to summarize the overall variability among individuals, which includes both the divergence between groups (*i.e.*, structured genetic variability), and the variation occurring within groups ('random' genetic variability). To assess the relationships between different clusters, an adequate method should focus on between-group variability, while neglecting within-group variation.

This is precisely the rationale of Discriminant Analysis (DA) [17,18]. This multivariate method defines a model in which genetic variation is partitioned into a between-group and a within-group component, and yields synthetic variables which maximize the first while minimizing the second (Figure 1). In other words, DA attempts to summarize the genetic differentiation between groups, while overlooking within-group variation. The method therefore achieves the best *discrimination *of individuals into pre-defined groups (Figure 1c). Interestingly, this method also allows for a probabilistic assignment of individuals to each group, as in Bayesian clustering methods.

Unfortunately, DA suffers from considerable restrictions which often preclude its application to genetic data. First, the method requires the number of variables (alleles) to be less than the number of observations (individuals). This condition is generally not fulfilled in Single Nucleotide Polymorphism (SNP) or re-sequencing datasets. Second, it is hampered by correlations between variables, which necessarily occur in allele frequencies due to the constant-row sum constraint [i.e., compositional data, [19,20]]. Moreover, the violation of the assumption of uncorrelated variables will be even more blatant in the presence of linkage disequilibrium. Therefore, the application of DA to genetic data has remained very limited so far [8,21].

In this paper, we introduce the Discriminant Analysis of Principal Components (DAPC), a new methodological approach which retains all assets of DA without being burdened by its limitations. DAPC relies on data transformation using PCA as a prior step to DA, which ensures that variables submitted to DA are perfectly uncorrelated, and that their number is less than that of analysed individuals. Without implying a necessary loss of genetic information, this transformation allows DA to be applied to any genetic data. Like PCA, our approach can be applied to very large datasets, such as hundreds of thousands of SNPs typed for thousands of individuals. Moreover, the contributions of alleles to the structures identified by DAPC can allow for identifying regions of the genome driving genetic divergence among groups. Along with the assignment of individuals to clusters, our method provides a visual assessment of between-population genetic structures, permitting to infer complex patterns such as hierarchical clustering or clines.

Whenever group priors are unknown, we use K-means clustering of principal components to identify groups of individuals [5,16]. K-means relies on the same model as DA to partition genetic variation into a between-group and a within-group component, and attempts to find groups that minimize the latter. Like in STRUCTURE, we run K-means clustering with different numbers of clusters, each of which gives rise to a statistical model and an associated likelihood. As advocated in previous studies [5,22], we use Bayesian Information Criterion (BIC) to assess the best supported model, and therefore the number and nature of clusters.

We apply DAPC to both simulated and empirical datasets. We use simulations to assess the ability of our approach to infer the right genetic clusters, and compare our results to those obtained with STRUCTURE [1,2]. Then, we illustrate the type of information that can be gathered by DAPC by applying the method to two empirical datasets. First, we analyse worldwide structuring of native human populations using the HGDP-CEPH cell line panel typed for microsatellite markers [23-25], enriched with additional populations of Native Americans [26]. Second, we use DAPC to study the temporal variation in seasonal influenza (H3N2) hemagglutinin (HA) segments from viruses collected in the northern hemisphere from 2001 to 2007. Both datasets, as well as the implementation of our methodology are available in the *adegenet *package [6] for the free software R [27].

## Results

### Analysis of simulated datasets

**Figure 2:**
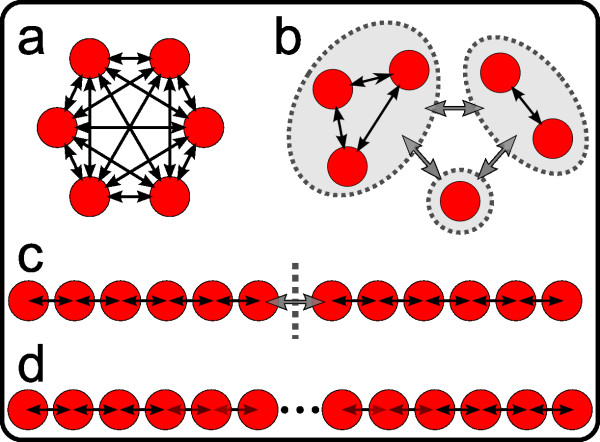
**Diagram of migration models used in simulations**. The four panels represent in (a) an island model, (b) a hierarchical island model, (c) hierarchical stepping stone, and in (d) a stepping stone with 24 populations. Red disks represent random mating sub-populations (demes) and arrows the interconnecting migration routes (black arrows represent greater gene flow than grey ones). Dotted lines indicate archipelagos (b) or a contact zone (c).

As a benchmark, we first compared the results of DAPC to those obtained by STRUCTURE using simulations. Data were simulated with EASYPOP [28] using four population genetic models (Figure 2): an island model (Figure 2a), a hierarchical islands model (Figure 2b), a one-dimensional hierarchical stepping stone (Figure 2c), and a standard one-dimensional stepping stone (Figure 2d). The number of populations varied from six for the island and hierarchical island models to 24 for the stepping stone model. Parameters of the simulations are provided in Table 1. They were chosen to ensure moderate genetic differentiation and realistic gene diversities (Table 2), and to reflect typical population genetic datasets for non-model organisms. All simulations were run for 3,000 generations. Inspection of summary statistics confirmed equilibrium had been reached in all simulations.

**Table 1:** Parameters of simulations.

	Island model	Hierarchical island model	Hierarchical stepping stone	Stepping stone
Number of populations	6	6 (3, 2, 1)	12 (6, 6)	24

Population size	200	200	100	50

Sample size^(1)^	100	100	50	25

Migration rate	0.005	0.05/0.005^(2)^	0.01/0.001^(2)^	0.02

Mutation rate	10^-4^	10^-4^	10^-4^	10^-4^

Number of loci	30	30	30	30

Possible allelic states	50	50	50	50

This table indicates the parameters used to simulate data under four different models (see Figure 2). ^(1)^Sample size refers to the number of individuals per population retained in the analyses.

^(2)^The first migration rate refers to between-population migration, whereas the second refers to migration between the higher hierarchical levels.

**Table 2:** Summary statistics of the simulations.

	Median	Quantile 5%	Quantile 95%
**Island model**			

*F*_ST_	0.1	0.07	0.13

*H*_S_	0.42	0.36	0.46

number of alleles/locus	5	3	8

**Hierarchical island model**			

*F*_ST_	0.05	0.03	0.08

*H*_S_	0.41	0.33	0.49

number of alleles/locus	5	2	8

**Hierarchical stepping stone**			

*F*_ST_	0.37	0.09	0.56

*H*_S_	0.3	0.2	0.38

number of alleles/locus	6	3	9

**Stepping stone**			

*F*_ST_	0.42	0.12	0.64

*H*_S_	0.27	0.13	0.36

number of alleles/locus	6	4	9

This table reports usual genetic summary statistics computed on the simulated datasets using *adegenet*. *F*_ST _refers to the mean pairwise *F*_ST _computed using Nei's estimator [62]. *H*_S _refers to the gene diversity (expected heterozygosity under random mating).

Ten independent replicates were obtained for each model. Each dataset was analysed by both STRUCTURE and DAPC. Accuracy of the results obtained with STRUCTURE depended critically on the underlying population genetic model behind the simulated data (Table 3). For the island model, STRUCTURE identified the true number of clusters in the majority of cases, and proved very efficient in assigning individuals to their actual group. In the hierarchical island model, STRUCTURE was less successful at identifying the actual number of subdivisions, while still providing accurate assignments. The performance decreased drastically in the two stepping stone models, where the method systematically failed to retrieve the true number of clusters. Moreover, even when enforcing STRUCTURE to partition individuals into the actual number of populations, the method largely failed to identify the existing groups.

**Table 3:** Results of the analyses of simulated data.

	Island Model	Hierarchical island model	Hierarchical stepping stone	Stepping stone
Number of populations (true *K*)	6	6	12	24

*K *inferred by DAPC	6 ([6,6])	6 ([6,8])	11 (8,12)	17.5 ([13,21])

*K *inferred by STRUCTURE	6 ([2,7])	3 ([2,6])	2 ([2,2])	2 ([2,5])

% of correct assignment by DAPC	98.2% ([96.3%,99%])	87.5% ([73.9%,91.2%])	89.7% ([87.9%,97.2%])	83.9% ([80%,88.7%])

% of correct assignment by STRUCTURE	98.6% ([98%,99.2%])	93.1% ([89.2%,95.5%])	NA^(1)^	NA^(1)^

This table reports the results of analyses of simulated data (see Figure 2) by DAPC and STRUCTURE. *K *refers to the number of clusters. Inferred numbers of clusters are reported as medians computed from the 10 replicates, with the range of variation provided within parentheses. ^(1)^NA is indicated when the percentage of successful assignment could not be computed with STRUCTURE. In these cases, the 'optimal' *K *was very different from the true *K*, resulting in meaningless assignments with numerous empty clusters and subsequently very low proportion of correct assignments.

**Figure 3:**
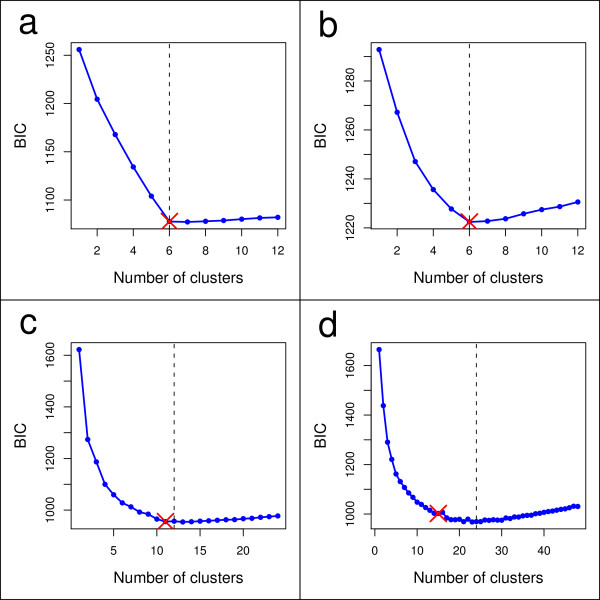
**Inference of the number of clusters in simulated data**. These four panel report examples of outputs from single simulations of the function *find.clusters *used to identify the number of clusters in data simulated according to for four different population genetics models (a: island model; b: hierarchical island model; c: hierarchical stepping stone and d: stepping stone; see Figure 2). Bayesian information criterion (BIC) is provided for different numbers of clusters. The chosen number of clusters is the minimum number of clusters after which the BIC increases or decreases by a negligible amount. The actual number of populations (*K*) is indicated by the dotted line.

The same datasets were analysed by DAPC using the *adegenet *package [6] for the R software [27]. The number of clusters was assessed using the function *find.clusters*, which runs successive K-means clustering with increasing number of clusters (*k*). We covered a wide range of possible clusters from one to 2*K*, where *K *was the actual number of demes in the simulations. Figure 3 illustrates the procedure for selecting the 'optimal' number of clusters. This choice was made on the basis of the lowest associated BIC (Figure 3a-b). In cases where the optimal number of clusters was ambiguous, *k *was increased as long as it resulted in a noticeable improvement in BIC (Figure 3c-d). Overall, this procedure recovered well the actual number of populations (Table 3). The number of clusters was always better inferred in island-based models (Figure 3a-b) than in more continuous population genetics models (Figure 3c-d), where clusters tend to dissolve into more clinal patterns of genetic differentiation. But even when the actual *K *was not identified, the inferred number of clusters generally remained relatively close to the true value (Table 3). Interestingly, the estimation of *K *by our method was markedly better than that achieved by STRUCTURE for all the studied models, including the classical island model for which our approach always inferred the exact number of clusters (Table 3). This result is consistent with previous studies which used K-means on principal components [5,16].

Then, DAPC was performed (function *dapc*) using clusters defined by K-means where we specified the actual number of clusters (*i.e.*, *k *= *K*). In all analyses, 50 principal components of PCA were retained in the data transformation step. The comparison of the final assignments of individuals to groups to the actual group memberships revealed that DAPC performed remarkably well. Assignment success varied depending on the population genetics model assumed in the simulations but remained high for all simulated datasets considered (Table 3). The frequency of correct assignments was highest in the island models, where DAPC performed essentially as well as STRUCTURE (Table 3). However, even in the stepping stone models (Figure 2c-d), successful assignment rates remained very satisfying, with correct assignment rates ranging from 80% to 97% depending on the replicate.

**Figure 4:**
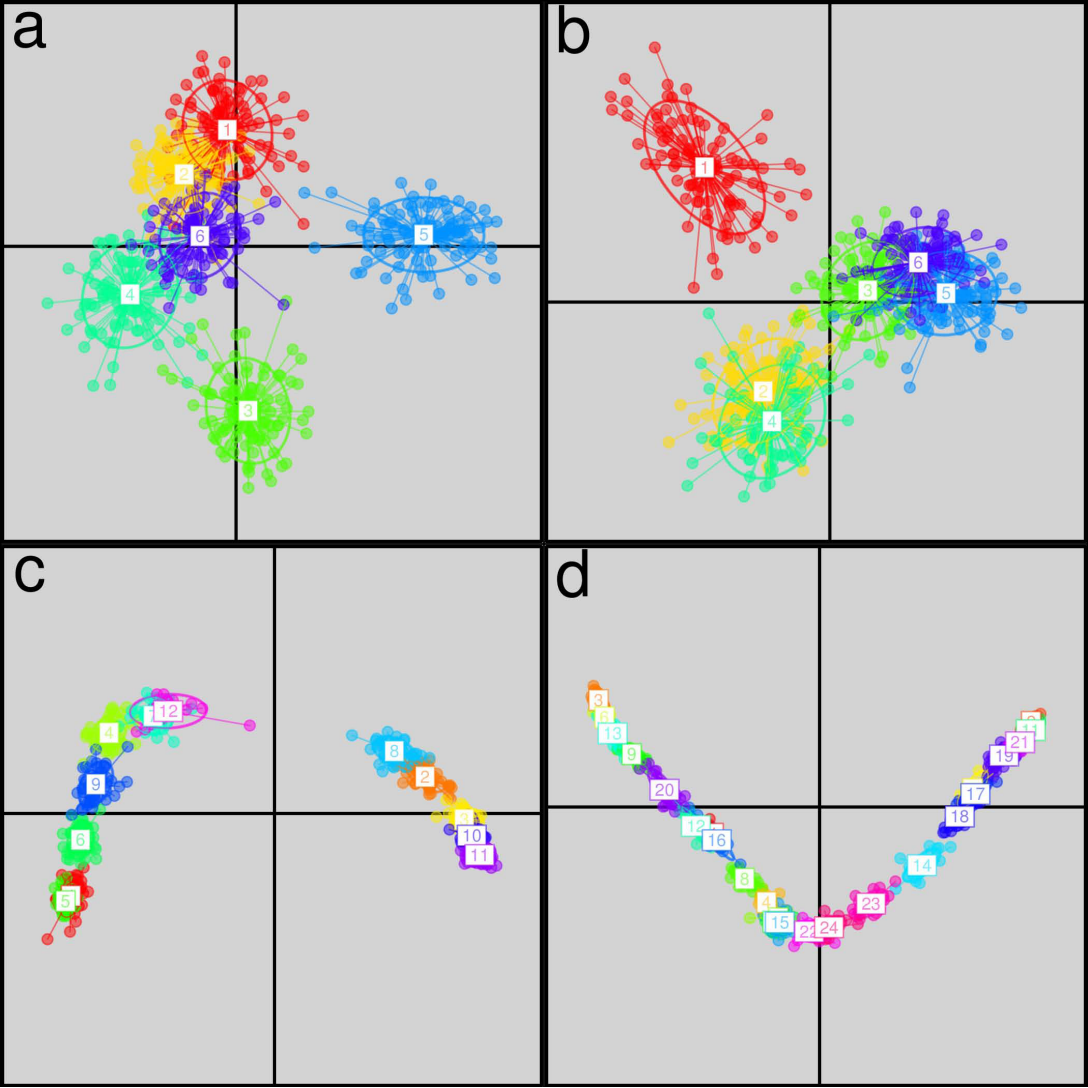
**Scatterplots of DAPC of simulated data**. These scatterplots show the first two principal components of the DAPC of data simulated according to four different models (a: island model; b: hierarchical islands model; c: hierarchical stepping stone and d: stepping stone; see Figure 2). Clusters are shown by different colours and inertia ellipses, while dots represent individuals.

Successful detection of the correct number of genetic clusters is undoubtedly a desirable feature. However, this information alone is not sufficient to describe the apportionment of genetic diversity within a population. What is additionally needed to gain real insights about the system under study is a representation of the relatedness between clusters. DAPC is particularly well suited for this task, as it finds principal components which best summarize the differences between clusters while neglecting within-cluster variation (Figure 1). The first principal components of DAPC can be plotted to obtain scatterplots, which provide a direct visual assessment of between-group structures (Figure 4). For instance, the hierarchical structure is clearly visible on Figure 4b, where three groups of genetically closer clusters can be identified ({1}, {2, 4}, and {3, 5, 6}). Results for the stepping stone model (Figure 4d) can easily be distinguished from the island model (Figure 4a) by the clinal arrangement of the clusters. And this model can in turn be distinguished from the hierarchical stepping stone, for which the scatterplot distinctly shows two separate clines (Figure 4c).

### Analysis of empirical data

#### Human microsatellite data

DAPC was applied to the microsatellite genotypes from the Human Genome Diversity Project-Centre d'Etude du Polymorphisme Humain (HGDP-CEPH) [23-25], an extensive dataset of native human populations distributed worldwide. This dataset was extended by adding genotypes from 24 Native American and Siberian populations [26]. The resulting dataset comprises 1350 individuals from 79 populations, genotyped for 678 microsatellite markers (8170 alleles).

**Figure 5:**
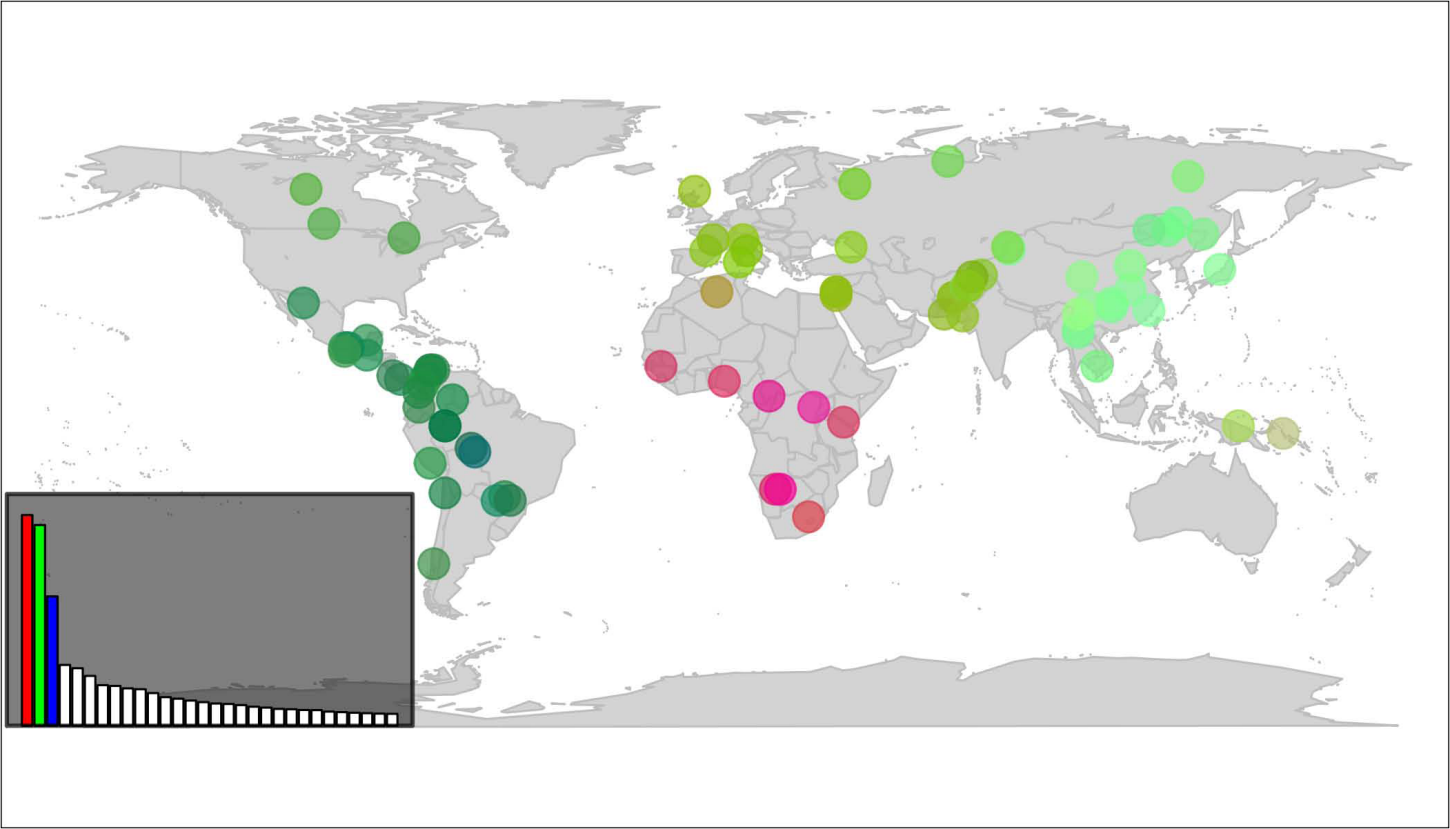
**Colorplot of the DAPC of extended HGDP-CEPH data**. This colorplot represents the first three principal components (PC) of the DAPC of extended HGDP-CEPH data, using populations as prior clusters. Each dot corresponds to a sampled population. Each principal component is recoded as intensities of a given colour channel of the RGB system: red (first PC), green (second PC), and blue (third PC). These channels are mixed to form colours representing the genetic similarity of populations. The inset indicates the eigenvalues of the analysis, with colour channels used to represent PCs indicated on the corresponding eigenvalues.

Two analyses were run for this dataset. First, we used DAPC to investigate the genetic structure of the 79 sampled populations. We retained 1,000 principal components of PCA during the preliminary variable transformation, which accounted for most (approximately 94%) of the total genetic variability. It is worth noting that despite the respectable size of this dataset (1350 individuals and 8170 alleles), DAPC was run in less than a minute on a standard desktop computer. The eigenvalues of the analysis (Figure 5, inset) showed that the genetic structure was captured by the first three principal components. These synthetic variables were mapped using colour coding to unravel patterns in the population structuring (Figure 5). The results obtained are remarkably clear and consistent with previous findings [25,26]. The first principal component (red channel, Figure 5) clearly differentiates Sub Saharan African populations from the rest of the world. The second principal component (green channel, Figure 5) displays a cline of genetic differentiation between Western Europe and East Asia. The third principal component (blue channel, Figure 5) highlights the differentiation of American populations from the rest of the world.

**Figure 6:**
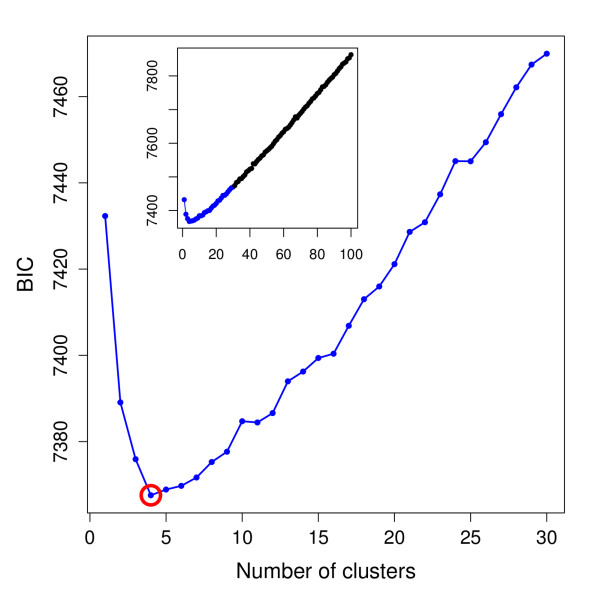
**Inference of the number of clusters in the extended HGDP-CEPH data**. This graph shows the output of the function *find.clusters *used to identify the number of clusters in extended HGDP-CEPH data. Bayesian information criterion (BIC) is provided for different numbers of clusters (from one to 100). The chosen number of clusters (4) is circled in red. The inset indicates the global results (up to 100 clusters), while the main figure shows the detail of the results up to 30 clusters.

**Figure 7:**
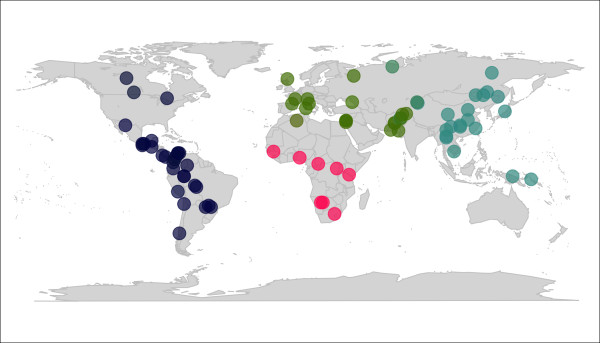
**Colorplot of the DAPC of extended HGDP-CEPH data based on four inferred clusters**. This colorplot represents the three principal components (PC) of the DAPC of extended HGDP-CEPH data, using the four clusters inferred by *find.clusters *(see Figure 6). Each dot corresponds to a sampled population. Each principal component is recoded as intensities of a given colour channel of the RGB system: red (first PC), green (second PC), and blue (third PC). These channels are mixed to form colours representing the genetic similarity of populations. Eigenvalues are not indicated, since there are only three PC in a DAPC based on four clusters.

While largely consistent with previous well-established findings, these results are based on the clustering of individuals into geographically predefined populations. This has the possible drawback that higher-level of genetic clustering could be overlooked. To evaluate this hypothesis, we looked for the best supported number of clusters using our approach based on K-means algorithm. Inspection of the BIC values ranging from one to 100 clusters clearly showed that a subdivision into four clusters should be considered (Figure 6). We then used DAPC to investigate the genetic structure of the four newly inferred groups. The resulting colorplot (Figure 7) defines clear-cut patterns which are strikingly similar to results previously obtained under a four clusters population genetics model with STRUCTURE [25,26,29].

#### Seasonal influenza (H3N2) hemagglutinin data

To illustrate the versatility of our approach, we selected a radically different dataset for the second example. We analysed the population structure of seasonal influenza A/H3N2 viruses using hemagglutinin (HA) sequences. Changes in the HA gene are largely responsible for immune escape of the virus (antigenic shift), and allow seasonal influenza to persist by mounting yearly epidemics peaking in winter [30-32]. These genetic changes also force influenza vaccines to be updated on a yearly basis. Influenza A virus genome is organized in eight segments analogous to chromosomes in eukaryotes. While exchanges of segments (genomic reassortment) occasionally happen during the replication of the virus in multiply infected hosts [30,33,34], we are unaware of evidences for within-segment recombination.

Assessing the genetic evolution of a pathogen through successive epidemics is of considerable epidemiological interest. In the case of seasonal influenza, we would like to ascertain how genetic changes accumulate among strains from one winter epidemic to the next. For this purpose, we retrieved all sequences of H3N2 hemagglutinin (HA) collected between 2001 and 2007 available from Genbank [35]. Only sequences for which a location (country) and a date (year and month) were available were retained, which allowed us to classify strains into yearly winter epidemics. Because of the temporal lag between influenza epidemics in the two hemispheres, and given the fact that most available sequences were sampled in the northern hemisphere, we restricted our analysis to strains from the northern hemisphere (latitudes above 23.4°north). DNA sequences and meta-information were retrieved from Genbank using *ad-hoc *R scripts. Alignments were obtained for a stretch of 990 bases using ClustalW [36] and further refined manually using Jalview [37]. Aligned sequences were then imported in R using the *ape *package [38], and SNPs were extracted from the sequences using *adegenet *[6]. The final dataset included 1903 strains characterized by 125 SNPs which resulted in a total of 334 alleles. All strains from 2001 to 2007 were classified into six winter epidemics (2001-2006). This was done by assigning all strains from the second half of the year with those from the first half of the following year. For example, the 2005 winter epidemic comprises all strains collected between the 1^st ^of July 2005 and the 30^th ^of June 2006.

**Figure 8:**
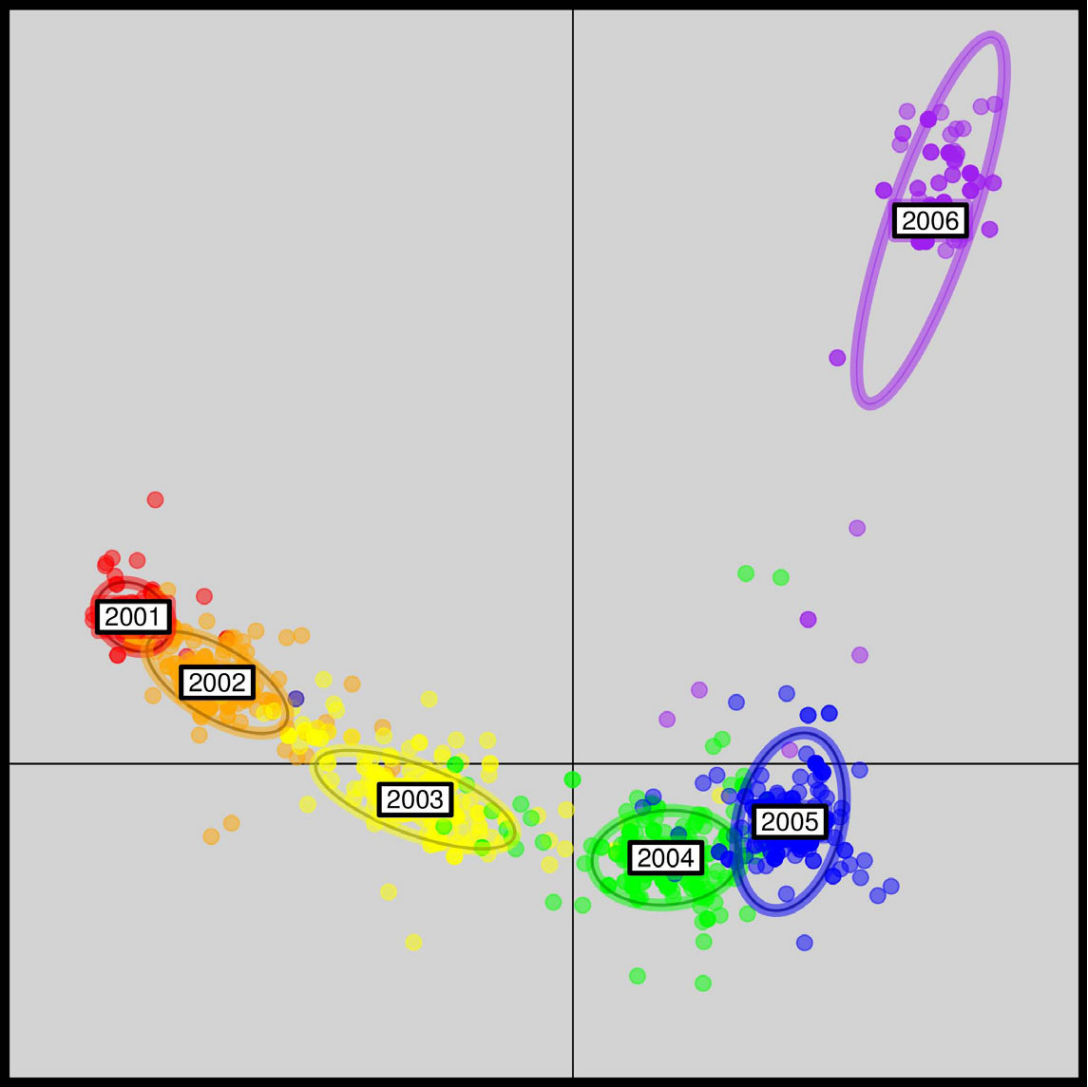
**Scatterplots of the DAPC of seasonal influenza (H3N2) data**. This scatterplot shows the first two principal components of the DAPC of seasonal influenza (H3N2) hemagglutinin data, using years of sampling as prior clusters. Groups are shown by different colours and inertia ellipses, while dots represent individual strains.

DAPC was used to investigate the pattern of genetic diversity in these data. We retained 150 principal components of PCA in the preliminary data transformation step, which altogether contained more that 90% of the total genetic variation. The first two principal components of DAPC were sufficient to summarize the temporal evolution of the virus (Figure 8). Epidemics appeared as clearly differentiated (Figure 8). Strains were correctly assigned to their winter epidemic in 92% of cases on average, with variation in correct assignment probabilities among epidemics ranging from 85% (2002) to 99% (2001). The first principal component of DAPC revealed the accumulation of genetic changes across epidemics, from 2001 to 2006 (Figure 8, horizontal axis). Interestingly, the 2006 epidemic was markedly isolated from the other epidemics on the second principal component (Figure 8, vertical axis), suggesting that more genetic changes had accumulated during 2005-2006 than during previous epidemics.

**Figure 9:**
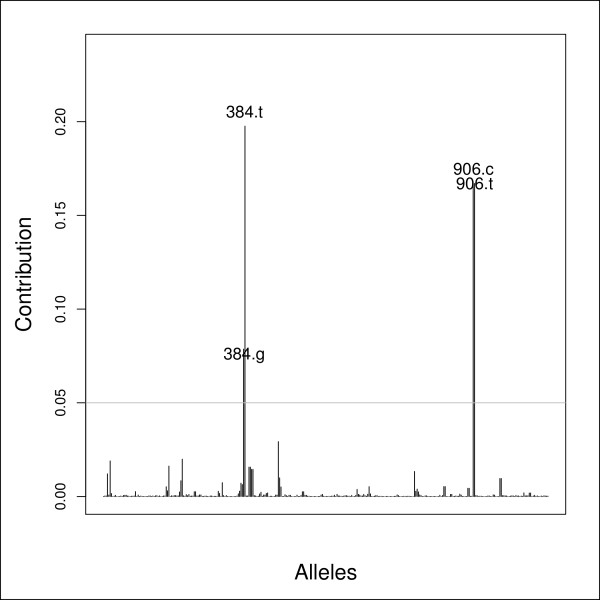
**Contributions of alleles to the second principal component of the DAPC of seasonal (H3N2) influenza data**. The height of each bar is proportional to the contribution (Equation 10) of the corresponding allele to the second principal component of the analysis, which isolated the strains from the 2006 influenza epidemic from all others (see Figure 8). Only alleles whose contribution is above an arbitrary threshold (grey horizontal line) are indicated for the sake of clarity. Alleles are labeled by their position in the original alignment, and the corresponding nucleotide, separated by a dot. Position 384 and 906 correspond respectively to residue 144 and 318 in the complete hemagglutinin (HA) protein CDS. Polymorphism at position 384 leads to a mutation from Asparagine to Lysine, present in 32.1% of strains sampled in 2006 while virtually absent before 2006. Polymorphism at position 906 is synonymous.

It has recently been suggested that seasonal influenza epidemics are seeded each year from a reservoir in Southeast Asia [31], from only a limited number of strains. This yearly seeding of epidemics leads to recurrent population bottlenecks and the marked differentiation of the 2006 epidemic may point to an unusually severe population bottleneck. Alternatively, this discontinuity might lie in some selective event affecting 2006 strains. To get some insight into the underlying causes of the differentiation of the 2006 epidemics, we inspected the associated allele loadings (Figure 9). The originality of the 2006 epidemics was largely driven by two SNPs coding for residue 144 and 318 in the HA protein with respective frequencies of 32.1% and 61.6% in 2006 but virtually absent in previous years. While such shifts in allele frequencies might be suggestive of natural selection, only one corresponded to a non-synonymous mutation from Asparagine to Lysine at position 144. Irrespective of the underlying mechanism driving the genetic isolation of the epidemics, DAPC dealt satisfyingly with the analysis of the influenza dataset by recovering the evolution over time of seasonal influenza strains, while also highlighting an interesting discontinuity between the 2005 and 2006 epidemics.

## Discussion and Conclusions

In this paper, we introduced a new multivariate method, the Discriminant Analysis of Principal Components (DAPC), for the analysis of the genetic structure of populations. This approach can be used to define clusters of individuals and to unravel possibly complex structures existing among clusters, such as hierarchical clustering and clinal differentiation, while being orders of magnitude faster than existing Bayesian clustering methods. For simulated data, DAPC proved as accurate as STRUCTURE in detecting hidden population clusters within simple island population models. Moreover, DAPC was more suited to unravel the underlying structuring in more complex population genetics models. Another major advantage of DAPC over Bayesian clustering approaches is the possibility to generate a graphical representation of the relatedness between the inferred clusters. Applied to two highly contrasted empirical datasets, our method was able to identify non-trivial and meaningful biological patterns.

One of the main assets of DAPC is its great versatility. Indeed, DAPC does not rely on a particular population genetics model, and is thus free of assumptions about Hardy-Weinberg equilibrium or linkage disequilibrium. As such it should be useful for a variety of organisms, irrespective of their ploidy and rate of genetic recombination. Also, contrary to Bayesian clustering methods, DAPC can be applied to very large datasets within negligible computational time (all analyses presented in this paper took less than minute to run on a standard computer). Moreover, the method is not restrained to genetic data, and can be applied to any quantitative data such as morphometric data. This feature is particularly interesting as it allows for partialling out the effects of undesirable covariates, such as different sequencing protocols, or trivial genetic structures that could obscure lesser, more interesting patterns. This can be achieved by analyzing the residuals of a preliminary model including the covariates as predictors instead of the raw data.

A major concern pertaining to all clustering approaches is the risk of inferring artefactual discrete groups in populations where genetic diversity is distributed continuously. Such spurious clusters are particularly likely to arise under spatially heterogeneous sampling of populations [39,40]. DAPC is not immune to this bias, and may indeed erroneously identify clusters within a cline. However, scatterplots provided by the method allow for a graphical assessment of the genetic structures between clusters (Figures 5 and 8), and provide remarkable insights as to how the genetic variability is organized. For instance, in our simulations based on stepping stone models (Figure 2c-d), DAPC clearly revealed the existence of clines (Figure 4c-d). Therefore, our approach is by no means restricted to the study of populations organised in discrete groups, and should be able to reveal more complex genetic patterns.

We chose to analyse two contrasted datasets to illustrate the versatility our approach. The HGDP-CEPH dataset has been repeatedly analysed using a variety of methods [29,39,41-47]. The DAPC results support previous evidence for discontinuities above and beyond the global clinal pattern in the apportionment of human genetic variation [29,43,48]. The subdivision inferred by DAPC is strikingly similar to the four clusters identified by the STRUCTURE software [25,26,29]. Note however, that the existence of large-scale clusters is not incompatible with a clinal distribution of genetic diversity and/or smaller-scale subdivisions [41,43]. These results illustrate that DAPC can be used as an efficient genetic clustering tool.

In contrast, the seasonal influenza analysis highlights features that go beyond simple genetic clustering. The DAPC scatterplot reveals that the virus is genetically structured into clusters which are arranged along a temporal cline, and shows a marked discontinuity between two successive years. Examination of allele loadings further reveals that this abrupt change is due to the apparition of new alleles in the global population, one of which induced a change in the amino-acid sequence, and may have therefore been subject to natural selection.

Although DAPC is a promising tool for the analysis of genetic data, further methodological developments should be considered to improve our approach. K-means has proved very efficient here as in previous studies for identifying genetic clusters [5], and is moreover consistent with the variance partition model used in Discriminant Analysis. However, this algorithm uses a very simple measure of group differentiation, and might struggle to identify the correct clusters in the most complex situations [16]. Would that be the case, useful alternatives to K-means could be found in more elaborated clustering algorithms [49]. Another point of interest relates to the selection of the number of principal components used in the prior dimension-reduction step. So far, this procedure is largely *ad hoc*, and relies on retaining most (more than 80%) of the genetic variance. Objective criteria would be useful to achieve this task. Unfortunately, there is no consensus on the best strategy for selecting interpretable principal components in PCA [50]. In the context of DAPC, we will have to evaluate a trade-off between the power of discrimination and the stability of assignments. Retaining more principal components provides more power for unravelling genetics structures, but increases the risks of obtaining *ad hoc *combinations of alleles which would discriminate perfectly the sampled individuals, whilst performing poorly on newly sampled individuals [51]. This issue could be addressed using repeated cross-validation, so that each individual would be assigned to a cluster based on a model calibrated using other individuals.

Irrespective of these methodological adjustments, we can see applications of DAPC beyond the mere study of the genetic structure of populations. One field where the method may be particularly relevant is association studies. In this context, population structuring ('*population stratification*') creates spurious correlations between genotypes and phenotypes. To circumvent this issue, Price et al. [12] proposed to partial out population structures by regressing data onto the first principal components of a PCA. But as explained in the introduction, PCA focuses on the overall variability, which includes variation between and within populations. In this case it would be preferable to remove only between-population structures from the data. Indeed, regression onto the first principal components of a PCA is likely to remove relevant within-population variation, thereby resulting in a lack of power for detecting significant associations. In contrast, DAPC yields principal components which are meant to reflect between-population variability only. Regressing data onto these synthetic variables would therefore remove the effects of population stratification, while preserving relevant variability. Note that one could achieve the same result by regressing data onto the groups identified by our approach.

Association studies aim at identifying genetic features that differ between two or more groups of individuals. In other words, the aim is to identify the alleles that best discriminate a set of pre-defined clusters. DAPC seems perfectly adapted to this task, as it finds linear combinations of alleles (the discriminant functions) which best separate the clusters. Alleles with the largest contributions to this discrimination are therefore those which are the most markedly different across groups, which could represent cases and controls. A simple plot of allele contributions (Figure 9) could therefore be used for a graphical assessment of alleles of major interest. An additional reason why DAPC may be well suited for this purpose is the ease with which one can control for covariates, such as age or sex.

To conclude, DAPC appears as a fast, powerful and flexible tool to unravel the makeup of genetically structured populations. However, we have no doubt that the application of this method goes way beyond the illustrations provided in this paper. We hope that its implementation in the free software R [27], which hosts an ever increasing number of tools for population genetics and phylogenetics [38,52-54] will open new and exciting perspectives for the statistical analysis of genetic data.

## Methods

### Measuring between-group differentiation

Discriminant Analysis (DA), DAPC, and K-means clustering all rely on the same statistical model to quantify between-group differentiation, which is in fact a classical ANOVA model. Below, we introduce this general model using concepts and notations further used in the specific presentation of DAPC and K-means clustering.

Let **y **∈ ℝ^*n *^be the vector of a centred variable with *n *observations (*y*_1_,...,*y*_*n*_) distributed into *g *groups, and **D **be the diagonal matrix containing uniform weights for the observations (*i.e.*, all diagonal entries are 1/*n*, while off-diagonal entries are 0). We denote **H = [***h*_*ij*_**] **the *n × g *matrix containing dummy vectors coding group membership, so that *h*_*ij *_= 1 if observation *i *belongs to group *j*, and *h*_*ij *_= 0 otherwise. We define **P = H(H**^*T*^**DH)**^**-1**^**H**^*T*^**D **as the projector onto the dummy vectors of **H**, which can be used to replace each observation in *y*_*i *_by the mean value of the group to which *i *belongs, ${\overset{\wedge }{y}}_{i}$. The ANOVA model relies on the decomposition of **y**:

(1) $$y=Py+(I-P)y=\overset{\wedge }{y}+\text{​}(y-\overset{\wedge }{y})$$

where **I **is the identity matrix of dimension *n*, $\overset{\wedge }{y}$ is the vector of predictions, and $\left(y-\overset{\wedge }{y}\right)$ is the vector of residuals. Since **y **is centred, the vectors $\overset{\wedge }{y}$ and $\left(y-\overset{\wedge }{y}\right)$ are also centred, and their squared norms (${\left\|y\right\|}_{D}^{2},{\left\|\overset{\wedge }{y}\right\|}_{D}^{2}$, and ${\left\|y-\overset{\wedge }{y}\right\|}_{D}^{2}$) equate their variances. Moreover, the Pythagorean theorem ensures that the total variance ($\mathrm{var}(y)={\left\|y\right\|}_{D}^{2}$) can be decomposed as:

(2) var(y)=b(y)+w(y)

where $b(y)={\left\|\overset{\wedge }{y}\right\|}_{D}^{2}$ is the variance between groups and $w(y)={\left\|y-\overset{\wedge }{y}\right\|}_{D}^{2}$ is the variance within groups. To measure the extent to which groups possess different values of **y**, we use the ratio of between-group and within-group variances, also known as the F statistic:

(3) $$\text{F}(y)=\frac{b(y)}{w(y)}$$

This quantity takes positive values only, with larger values indicating stronger differences between groups. Alternatively, one could use the proportion of variance explained by the model, which is also known as the *correlation ratio *of **y**, defined as:

(4) $$\eta^{2}(y)=\frac{b(y)}{\mathrm{var}(y)}$$

In fact, both quantities can be used as a measure of group separation in DA and DAPC, and would yield identical results (discriminant functions) up to a constant. In the remaining, we shall refer to the F statistic only.

### Discriminant Analysis of Principal Components

Let **X **be a *n × p *genetic data matrix with *n *individuals in rows and *p *relative frequencies of alleles in columns. For example, in the case of a locus with three alleles (A_1_, A_2_, A_3_), a homozygote genotype A_1_/A_1 _is coded as [1, 0, 0], while a heterozygote A_2_/A_3 _is coded as [0, 0.5, 0.5]. We denote **X**^*j *^the *j*^th ^allele-column of **X**. Missing data are replaced with the mean frequency of the corresponding allele, which avoids adding artefactual between-group differentiation. Without loss of generality, we assume that each column of **X **is centred to mean zero. Classical (linear) discriminant analysis seeks linear combinations of alleles with the form:

(5) $$f(v)=\sum_{j=1}^{p}X^{j}v_{j}=Xv$$

(**v **= [*v*_1_...*v*_*p*_]^*T *^being a vector of *p *alleles loadings, known as '*discriminant coefficients*'), showing as well as possible the separation between groups as measured by the F statistic (Equation 3). That is, the aim of DA is to choose **v **so that **F(Xv) **is maximum.

Linear combinations of alleles (Equation 5) optimizing this criterion are called *principal components*, which in the case of the discriminant analysis are also called *discriminant functions*. Discriminant functions are found by the eigenanalysis of the **D**-symmetric matrix [51]:

(6) $$PX{\left(W\right)}^{-1}X^{T}P^{T}D$$

where **P **is the previously defined projector onto the dummy vectors of **H**, and **W **is the matrix of covariances within groups, computed as:

(7) $$W=X^{T}{\left(I-P\right)}^{T}D(I-P)X$$

This solution requires **W **to be invertible, which is not the case when the number of alleles *p *is greater than the number of individuals *n*. Moreover, this inverse is numerically unstable ('ill-conditioned') whenever variables are correlated, which is always the case in allele frequencies and can be worsened by the presence of linkage disequilibrium.

To circumvent this issue, DAPC uses a data transformation based on PCA prior to DA. Rather than analyzing directly **X**, we first compute the principal components of PCA, **XU**, verifying:

(8) $$X^{T}DXU=U\Lambda$$

where **U **is a *p *× *r *matrix of eigenvectors (in columns) of **X**^*T*^**DX**, and **Λ **the diagonal matrix of corresponding non-null eigenvalues. Note that when the number of alleles (*p*) is larger than the number of individuals (*n*), we can alternatively proceed to the eigenanalysis of **XX**^*T*^**D **to obtain **U **and **Λ **[55], which can save considerable computational time. By definition, the number of principal components (*r*) cannot exceed the number of individuals or alleles (*r *≤ min(*n*, *p*)), which solves the issue relating to the number of variables used in DA. Moreover, principal components are, by construction, uncorrelated, which solves the other issue pertaining to the presence of collinearity among allele frequencies.

DA is then performed on the matrix of principal components. At this step, less-informative principal components may be discarded, although this is not mandatory. Replacing **X **with **XU **into Equation 6, the solution of DAPC is given by the eigenanalysis of the **D**-symmetric matrix:

(9) $$PXU{\left(U^{T}WU\right)}^{-1}U^{T}X^{T}P^{T}D$$

The first obtained eigenvector **v **maximizes *b*(**XUv**) under the constraint that *w*(**XUv) **= 1, which amounts to maximizing the F-statistic of **XUv**. This maximum is attained for the eigenvalue *γ *associated to **v **(*i.e.*, **F(XUv) **= *γ*). In other words, the loadings stored in the vector **v **can be used to compute the linear combinations of principal components of PCA (**XU**) which best discriminate the populations in the sense of the F-statistic.

However, it can be noticed that these linear combinations of principal components ((**XU**)**v**) can also be interpreted as linear combinations of alleles (**X**(**Uv**)), in which the allele loadings are the entries of the vector **Uv**. This has the advantage of allowing one to quantify the contribution of a given allele to a particular structure. Denoting *z*_*j *_the loading of the *j*^th ^allele (*j *= 1,...,*p*) for the discriminant function **XUv**, the contribution of this allele can be computed as:

(10) $$\frac{z_{j}^{2}}{\sum_{j=1}^{p}z_{j}^{2}}$$

### Prior clustering using K-means

Whenever groups are not known in advance, it is possible to define them using a clustering algorithm. K-means is a natural choice to do so since it uses the same model as DA and a similar measure of group differentiation. K-means relies on the model in equation (1) which decomposes the total variance of a variable into between-group and within-group components. This model can be extended to the multivariate case by summing variance components over the different variables. To differentiate univariate and multivariate variances, we use upper case notations for variances of multivariate data. Note, however, that these quantities are in both cases squared norms of vectors or matrices (considering the Frobenius norm in the multivariate case). Applied to the previously-defined matrix of principal components of PCA (**XU**) as in [5,16], this model can be written:

(11) VAR(XU)=B(XU)+W(XU)

with VAR(**X**) = tr(Λ), *B*(**XU**) = tr(**U**^*T*^**X**^*T*^**P**^*T*^**DPXU**), and *W***(XU) **= tr(**U**^*T*^**WU**). The Bayesian Information Criterion (BIC) used to choose the best clustering model is then defined as:

(12) BIC=nlog(W(X))+glog(n)

where *W***(X) **is the residual variance (*i.e.*, variance within groups, Equation 2) and *g *is the number of groups. This criterion quantifies the lack of fit of the model, while penalising the number of clusters used. Note that here, *g *is used as an *ad hoc *way of avoiding overfitting, and does not estimate the parametric dimensionality of the model as in the original formulation of BIC [56]. Several K-means can be run separately with different numbers of groups, and the best runs can be inferred from the decrease of BIC. In simulated data, BIC proved more efficient for identifying the correct number of clusters than other criteria such as Akaike Information Criterion (AIC) or the adjusted R^2 ^(results not shown). This result is consistent with previous findings which advocated the use of BIC for selecting the best number of groups in K-means clustering of genetic data [5].

### Clustering analyses using STRUCTURE

We used STRUCTURE [1,2] as a benchmark for the performance of DAPC. We analysed all simulated datasets with STRUCTURE v2.1, using the admixture model with correlated allele frequencies to determine the optimal number of genetic clusters and to assign individuals to groups. Computations were performed on the computer resources of the Computational Biology Service Unit at Cornell University (http://cbsuapps.tc.cornell.edu/). For each run, results were based on a Markov Chain Monte Carlo (MCMC) of 100,000 steps, of which the first 20,000 were discarded as burn-in. Analyses were ran with numbers of clusters (*k*) ranging from 1 to 8 for the island and hierarchical island models (Figure 2a-b), from 1 to 15 for the hierarchical stepping stone (Figure 2c), and from 1 to 30 for the stepping stone (Figure 2d). Ten runs were performed for each *k *value. We employed the approach of Evanno *et al. *[57] to assess the optimal number of clusters. In order to assess assignment success, STRUCTURE was run by enforcing *k *to its true value. Individuals were assigned to clusters using CLUMPP 1.1.2 [58], which allows to account for the variability in individual membership probabilities across the different runs. To obtain results comparable to DAPC, individuals were assigned to the cluster to which they had the highest probability to belong.

### Implementation and examples

The methodological approach presented in the paper is implemented in the *adegenet *package [6] for the R software [27]. The function *find.clusters *runs successive K-means for a range of *k *values, and computes the BIC of the corresponding models. The basic K-means procedure is implemented by the function *kmeans *in the *stats *package [27]. DAPC is implemented as the function *dapc*, and relies on procedures from *ade4 *[55,59,60] and *MASS *[61] to perform PCA (*dudi.pca*) and DA (*lda*). Both *find.clusters *and *dapc *can be used with any quantitative data, and have specific implementations for genetic data. The analysis of the four simulated datasets presented in Figures 4 and 5 can be reproduced by executing the example of the dataset *dapcIllus*. Similarly, analyses of the extended HGDP-CEPH and of the seasonal influenza (H3N2) data can be reproduced by executing the example of the datasets *eHGDP *and *H3N2, *respectively. Documentation and support can be found at the *adegenet *website (http://adegenet.r-forge.r-project.org/).

## Authors' contributions

TJ developed and implemented the method. FB performed the simulations. All authors contributed to analyzing and interpreting the data, and to writing the manuscript. All authors read and approved the final manuscript.

## Authors' informations

TJ is a post-doctoral research associate in biometry at the Imperial College London, UK. His main focus is on developing statistical tools for analysing genetic data, with an emphasis on multivariate methods. FB is an associate professor in population genetics at the Imperial College London, UK. His work ranges from theoretical to applied population genetics, with an emphasis on Human populations and their pathogens. SD is an assistant professor in evolutionary biology and biostatistics at the Université Claude Bernard - Lyon 1, France. His interests range from empirical studies to theoretical works in population biology, ecology, and evolution.
